# Giving voice to the end-user: input on multipurpose prevention technologies from the perspectives of young women in Kenya and South Africa

**DOI:** 10.1080/26410397.2021.1927477

**Published:** 2021-07-05

**Authors:** Alexandra M. Minnis, Emily Krogstad, Mary Kate Shapley-Quinn, Kawango Agot, Khatija Ahmed, L. Danielle Wagner, Ariane van der Straten, on behalf of the TRIO Study Team

**Affiliations:** a Senior Research Epidemiologist and Director, Women's Global Health Imperative, RTI International, Berkeley, CA, USA; Associate Adjunct Professor, School of Public Health, University of California, Berkeley, CA, USA. *Correspondence*: aminnis@rti.org; b Scholar, Desmond Tutu HIV Centre, University of Cape Town, Cape Town, South Africa; c Project Manager, Women's Global Health Imperative, RTI International, Berkeley, CA, USA; d Executive Director, Impact Research and Development Organization, Kisumu, Kenya; e Chief Executive Officer, Setshaba Research Centre, Soshanguve, South Africa; f Project Coordinator, Women's Global Health Imperative, RTI International, Berkeley, CA, USA; g Senior Fellow, Women's Global Health Imperative, RTI International, Berkeley, CA, USA; Adjunct Professor, Center for AIDS Prevention Studies, Department of Medicine, University of California, San Francisco, CA, USA; Consultant, ASTRA Consulting, Kensington, CA, USA

**Keywords:** multipurpose prevention technologies (MPTs), contraceptive preferences, end-user, young women

## Abstract

Unintended pregnancy and unmet need for modern contraception contribute substantially to reproductive health disparities globally. In sub-Saharan Africa they occur in contexts of disproportionately high rates of HIV infection. Multipurpose prevention technologies (MPTs) can address HIV and pregnancy prevention needs in a single “2-in-1” product; however, few studies have solicited end-user views to inform design of new MPTs. We conducted the Tablets, Ring, Injections as Options (TRIO) study with young women aged 18−30 in Kenya and South Africa (*N* = 277) to examine preferences and acceptability of future MPTs. In a randomised clinical cross-over study in which women used three placebo delivery forms, we complemented quantitative acceptability assessments with in-depth interviews and focus group discussions (*N* = 88 participants). We examined anticipated enablers and barriers to adoption and use of future MPTs and synthesised novel product design recommendations. Participants expressed high interest in MPTs. Anticipated side effects constituted a primary concern; however, many expected barriers were not dosage form-specific, but addressed contextual factors instead, such as fears regarding use of new biomedical technologies, misunderstandings and stigma regarding use, and navigating partner disclosure and engagement. Women preferred MPTs that offered discreetness and long-duration protection to minimise user-burden, did not interfere with their relationships, and conferred protection for unanticipated situations. End-user research to identify and pre-emptively address potential barriers while underscoring benefits to a new MPT product is vital. Attention to cultural contexts in implementation of new MPTs is important to communicating perceived benefits, achieving acceptability and maximising public health benefits.

## Résumé

Les grossesses non désirées et les besoins insatisfaits de méthodes modernes de contraception contribuent substantiellement aux disparités de santé reproductive dans le monde. En Afrique subsaharienne, ces disparités se produisent dans des contextes de taux anormalement élevés d’infection à VIH. Les technologies polyvalentes de prévention (TPP) peuvent répondre aux besoins en matière de prévention du VIH et de grossesses non désirées avec un produit unique « deux en un »; néanmoins, peu d’études ont demandé l‘avis des utilisateurs finals pour guider la conception des nouvelles TPP. Nous avons réalisé une étude sur les comprimés, les anneaux et les injections comme options (étude TRIO) auprès de jeunes femmes âgées de 18 à 30 ans en Afrique du Sud et au Kenya (n = 277) pour examiner les préférences et l’acceptabilité de futures TPP. Au cours d’une étude croisée clinique randomisée dans laquelle les femmes utilisaient trois modes de fourniture de placebos, nous avons complété des évaluations quantitatives de l’acceptabilité par des entretiens approfondis et des discussions de groupes d’intérêt (n = 88 participantes). Nous avons analysé les facteurs habilitants et les obstacles envisagés à l’adoption et l’utilisation de futures TPP, puis synthétisé des recommandations sur la conception de nouveaux produits. Les participantes ont exprimé un vif intérêt pour les TPP. Les effets secondaires prévus représentaient une préoccupation majeure; néanmoins, beaucoup d’obstacles escomptés n’étaient pas spécifiques à la forme du dosage, mais avaient plutôt trait à des facteurs contextuels, comme les craintes concernant l’utilisation de nouvelles technologies biomédicales, les idées erronées et la stigmatisation liée à l’utilisation des méthodes, ou se rapportaient au processus de divulgation au partenaire et à sa participation. Les femmes préféraient les TPP qui garantissaient la discrétion et une protection de longue durée pour minimiser la charge de l’utilisation, mais sans interférer avec leurs relations, et qui conféraient une protection dans des situations imprévues. La recherche auprès des utilisatrices finales pour identifier les obstacles potentiels et s’y attaquer de manière préventive tout en mettant en évidence les avantages d’un nouveau produit de TPP est capitale. Il est important d’accorder une attention aux contextes culturels dans la mise en œuvre de nouvelles TPP pour communiquer les avantages perçus, parvenir à l’acceptabilité et maximiser les avantages de santé publique.

## Resumen

El embarazo no intencional y la necesidad insatisfecha de anticoncepción moderna contribuyen considerablemente a las disparidades en salud reproductiva a nivel mundial. En África subsahariana, ocurren en contextos con tasas desproporcionadamente altas de infección por VIH. Las tecnologías de prevención multipropósito (MPT) pueden atender las necesidades de prevención de VIH y embarazo en un solo producto “2 por 1”; sin embargo, pocos estudios han solicitado los puntos de vista de usuarias finales para informar el diseño de nuevas MPT. Realizamos el estudio de Tabletas, Anillo, Inyecciones como Opciones (TRIO) con mujeres jóvenes de 18 a 30 años en Kenia y Sudáfrica (N = 277) para examinar las preferencias y aceptabilidad de futuras MPT. En un estudio clínico cruzado aleatorizado donde las mujeres utilizaron tres formas de administración de placebo, suplementamos las evaluaciones cuantitativas de aceptabilidad con entrevistas a profundidad y discusiones en grupos focales (N = 88 participantes). Examinamos barreras y facilitadores previstos con relación a la adopción y el uso de futuras MPT y sintetizamos recomendaciones sobre el diseño de productos novedosos. Las participantes expresaron gran interés en las MPT. Los efectos secundarios previstos constituían una preocupación principal; sin embargo, muchas barreras esperadas no estaban relacionadas específicamente con la forma de administración, sino que abordaban factores contextuales, tales como temores sobre el uso de nuevas tecnologías biomédicas, malentendidos y estigma relacionado con el uso, así como navegar por la divulgación y participación de parejas. Las mujeres prefirieron las MPT que ofrecían discreción y protección de larga duración para minimizar la carga de la usuaria, que no interferían con sus relaciones y que conferían protección para situaciones imprevistas. Para identificar y abordar de manera preventiva las posibles barreras y a la vez subrayar los beneficios de un nuevo producto de MPT, es vital realizar investigaciones con usuarias finales. La atención a los contextos culturales en la aplicación de nuevas MPT es importante para comunicar beneficios percibidos, lograr aceptabilidad y maximizar los beneficios para la salud pública.

## Background

Women’s need for effective and affordable contraception remains a worldwide priority, particularly in low-resource settings where populations experience the highest fertility rates, lowest utilisation of contraceptives and highest rates of maternal and child mortality.^1,2^ Unmet need for contraceptives, defined as women who would like to avoid pregnancy but are not using and/or are unable to access a modern contraceptive method, contributes substantively to health disparities. Globally, 214 million reproductive-age women in developing regions are estimated to have an unmet need for modern contraception.^3^ Sub-Saharan Africa accounts for 53 million women with unmet need and has the highest total fertility rate (4.9 births per woman).^3^ The United Nations 2030 Agenda for Sustainable Development articulates goals for achieving “healthy lives and promoting well-being.”^4^ Reducing unintended pregnancy through access to modern contraceptives constitutes a key strategy to achieving this goal.^4^

High rates of unintended pregnancy and unmet need for contraception occur in a context of disproportionately high rates of HIV infection, particularly for adolescents and young women.^5–8^ The Evidence for Contraceptive Options and HIV Outcomes (ECHO) trial, which established that HIV risk did not differ significantly across three highly effective, reversible, contraceptive methods in comparison to one another, also revealed high HIV, chlamydia and gonorrhoea incidence despite a comprehensive HIV prevention package.^9,10^ The ECHO team underscored the urgent need to pursue integration of pregnancy and HIV prevention services to meet the dual prevention needs of women across sub-Saharan Africa. Multipurpose prevention technologies (MPTs) are an innovative class of products that are uniquely suited to answer this call.

MPTs are an innovative group of prevention products that offer protection against at least two sexual and reproductive health risks, including HIV, other sexually transmitted infections (STIs), and unintended pregnancy. While condoms are the only MPT currently available, a number of MPTs, across a range of delivery forms, are in the product development pipeline.^11^ Vaginally delivered, on-demand products used prior to intercourse (e.g. gels, fast-dissolving inserts, films) comprise one focus of the current MPT pipeline. Longer-acting approaches are likewise in various stages of research and development. MPT vaginal rings offering continuous use (e.g. one or three months duration) constitute the delivery form with the greatest number of products in development currently.^12^ Vaginal rings containing both antiviral and contraceptive agents to offer combined HIV and pregnancy prevention, for example, have been tested in recent clinical trials conducted in the United States and Kenya to evaluate safety, pharmacokinetics, tolerability and acceptability.^13,14^ Other delivery forms, such as long-acting MPT implants,^15^ are in pre-clinical development and may offer co-delivered (i.e. single insertion, with potential for retrieval of one indication) and co-formulated products.

Nevertheless, it remains critical to ensure that new MPTs are designed in alignment with end-user preferences and implemented in strategic ways to maximise effectiveness and public health benefit.^16^ Introduction of existing prevention methods, alongside research with novel products in development, has highlighted the importance of product choice throughout different life stages and the varied and dynamic needs of different user groups and individuals. A method mix that ultimately offers choice to end-users is recognised as critical to realising public health goals of reducing rates of HIV infection and unintended pregnancy. Here we share perspectives from young Kenyan and South African women who had actual experience using three different placebo MPT delivery forms (monthly vaginal ring, daily oral tablet and monthly double gluteal injections) as part of the TRIO clinical study.^17–19^ The study was motivated by evidence that engaging and empowering end-users to shape future HIV prevention options is a critical step to achieving high adoption and use of novel prevention tools downstream. These women speak uniquely to the advantages, disadvantages and potential acceptability of various MPT attributes, with perspectives based on personal experiences with each TRIO product. We examined anticipated barriers to adoption and use of future MPT, preferred product characteristics and novel product design recommendations.

## Methods

### Study setting and design

**Figure 1.**
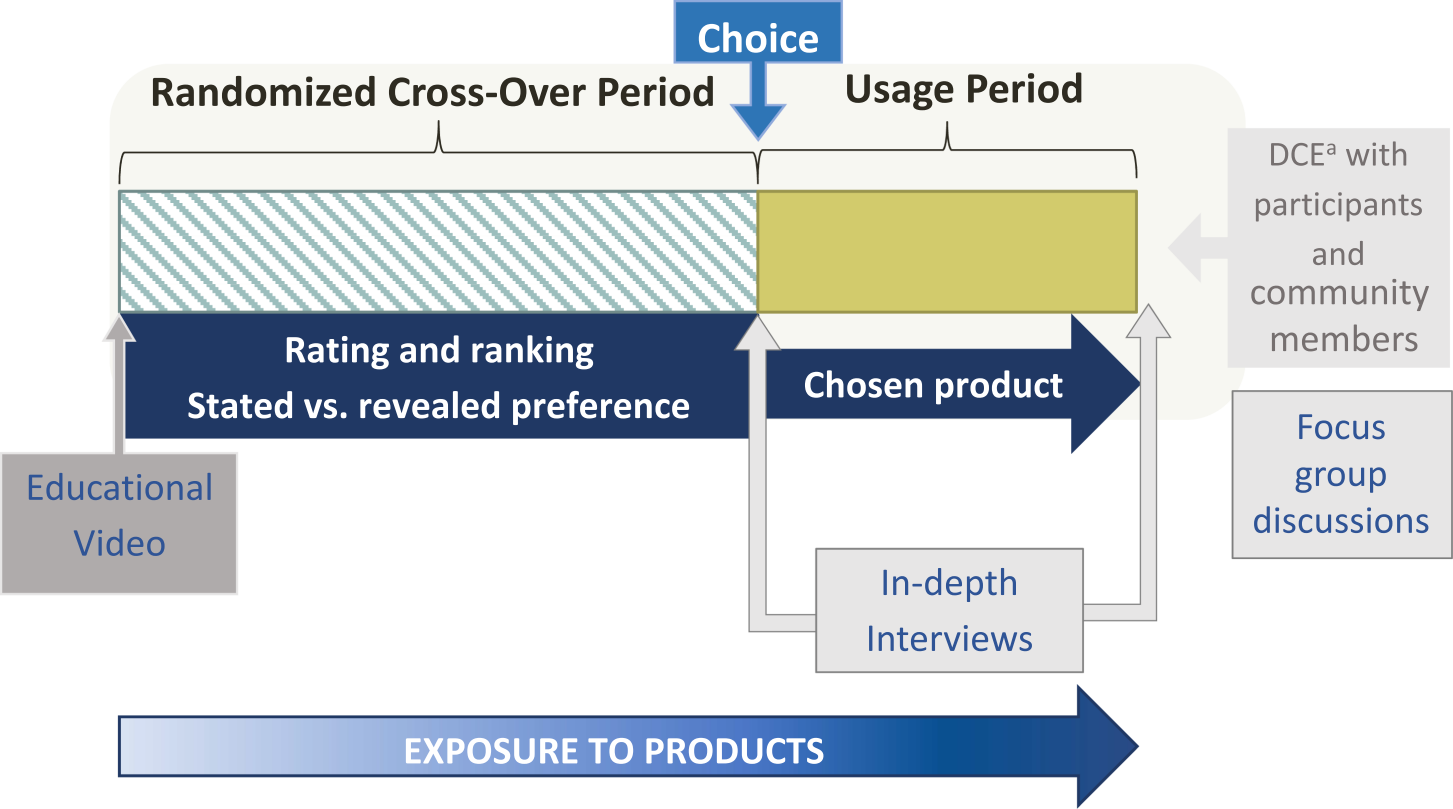
TRIO study design overview Note: TRIO evaluated three products during the cross-over period, with women randomized to a sequence in which they used each product for one month. Women chose their preferred product to use during the subsequent 2-month usage period. A subset of women completed in-depth interviews at the end of each study period. Focus group discussions were held at the end of the study.^a^DCE = discrete choice experiment.

The TRIO study took place between 2015 and 2017 in Kenya and South Africa and was conducted in partnership with two local research organisations: Impact Research and Development Organization (IRDO), in Kisumu, Kenya and Setshaba Research Centre (SRC), in Soshanguve, South Africa. Both settings experience high HIV prevalence and incidence. In Kisumu, HIV prevalence among adults aged 15–49 was estimated to be 17.3% in 2019, compared with 4.5% in Kenya, overall.^20^ Soshanguve, located in Gauteng Province, had an estimated 2017 HIV prevalence of 17.6%.^21^ Study procedures have been described in detail elsewhere and the study design is presented in Figure 1.^17–19,22–24^ Participants were recruited from urban and peri-urban communities surrounding each research site. Prior to eligibility screening visits, our teams held introductory engagement workshops with women (group size ranged from 15 to 30) to frame the study as an opportunity to contribute as a “co-designer” in MPT product development. Workshop content framed study participants as a key source of feedback for product developers and underscored the value of honest feedback on the products, including dislikes and negative experiences.

TRIO used a randomised, cross-over design to evaluate the acceptability of three drug delivery forms for HIV and pregnancy prevention: daily oral tablets, a monthly vaginal ring and monthly double injections (one in each gluteus). All products were placebos, providing a focus on attributes of the delivery forms themselves, free from drug-related side effects and varying (or unknown) efficacy that might influence acceptability. During the first stage of the study, participants used each of the products for one month in a randomised sequence (cross-over period). They were then asked to select one product to use for the subsequent two months (stage 2, usage period). At monthly study visits, participants completed interviewer-administered behavioural questionnaires, had clinical visits with guidance on using each product, and received condoms and risk-reduction counselling. At the last visit, participants completed an interviewer-administered questionnaire assessing preferences for products that prevent HIV and pregnancy. Participants were females, aged 18−30, sexually active, HIV-negative, not pregnant, and microbicide and PrEP naïve. Ethical approval for the study was obtained from Pharma-Ethics in South Africa (#150110905) and the KEMRI Scientific and Ethics Review Unit in Kenya (#KEMRI/RES/7/3/1: protocol #474). All participants provided written informed consent for inclusion in the study.

To complement the clinical study data collection, we conducted qualitative research activities at the conclusion of each study stage. All qualitative data collection was directed by semi-structured interview guides and conducted by trained social science interviewers. At the end of the cross-over period, a subset of participants was randomly selected (balanced by site) to complete an in-depth interview (IDI). This interview explored factors influencing product preferences, acceptability and use, with attention to discussion of each of the three TRIO delivery forms and contextual influences on women’s opinions and experiences with trying them.^24^ At the end of stage 2, we conducted an additional set of IDIs with participants who switched products during this period. These interviews used a similar guide to the earlier set of IDIs, with the addition of questions pertaining to switching products and extended inquiry regarding MPT preferences. We also convened six focus group discussions (FGDs) (three per site with one group for each product) at the end of the study with purposively selected women, such that each group was comprised of women who had selected the same TRIO product for use during stage 2. The FGD guide focused on acceptability and product preferences of the three product forms used during TRIO and ascertained views regarding anticipated preferences of women in their communities regarding MPTs.

### Analysis

The codebook development process for this data was concept-driven, based on codebooks used in similar studies, a conceptual model of HIV prevention product acceptability,^25^ and preliminary reviews of transcripts. All transcripts were coded in Dedoose by a team of five coders who met regularly and maintained high inter-rater reliability, iteratively modifying the codebook to capture emerging themes throughout the coding process.^24^ Here, we used content analysis encompassing two processes. First, we compiled a code report of all the IDI and FGD data where the code “MPT” was applied. The MPT code captured excerpts of IDIs and FGDs that specifically discussed: “different MPTs and their administration, including products not evaluated in TRIO: e.g. implants, vaginal gels, inserts, films”. One analyst (EK) with a background in bioengineering and end-user research reviewed this code report and synthesised all relevant excerpts into a single summary memo on themes related to participant views on MPT products. Second, given MPT-focused questions in IDIs (stage 2) and FGDs, those 15 transcripts were reviewed separately in full to capture discussions related to MPT products that may have not been captured with a more deductive use of the “MPT” code. In this process, we used a matrix framework to summarise key themes, which included: barriers to MPT use, including fears, misconceptions and relational effects, as well as suggestions for attributes, new dosage forms and use preferences for future MPTs. All themes generated in the first and second process (summary memo and matrix framework) were discussed by a team of two researchers (EK and AM) for validation; syntheses were then reviewed by other members of the TRIO team who had analysed the qualitative data with other research objectives.

Counts and frequencies were used to summarise quantitative data. Chi-squared tests were used to test for differences by country and by those who participated in the qualitative portion of the study.

## Results

### Study population

The TRIO clinical study enrolled 277 women and 246 (89%) completed the study. Approximately one-third participated in a qualitative study activity: 55 women completed IDIs and 37 women participated in the 6 FGDs (4 women completed both). Demographics of TRIO’s qualitative sub-sample have been described previously^24^; however, key sociodemographic characteristics for the overall clinical sample and the qualitative sub-sample are presented in Table 1. Participants had a median age of 23 years. At enrolment, 89% reported current contraceptive use, with injection (42%) and male condom (51%) use most common. Most women (70%) had ever used injectable contraceptives (more common in South Africa (82%) than Kenya (59%), *p *< 0.001); about one-third (35%) had previously used contraceptive implants (more common in Kenya than South Africa – 40% vs. 24%) and 5% had used intrauterine devices (IUDs). One-third of participants (35%) reported dual method use currently (condoms and another contraceptive method), with 23% reporting dual method use at last sex. Other characteristics of South African women differed from those in Kenya. Very few South African participants were married or cohabitating (9%), compared with 48% of Kenyan participants (*p *< 0.001). More South African than Kenyan participants had completed secondary school (61% vs. 42%, *p* = 0.001).

**Table 1.** Characteristics of women enrolled in the TRIO study (2015–2017), and the sub-sample of women selected to participant in a qualitative activity, by country

	Qualitative sub-sample						TRIO study sample	
	Soshanguve, South Africa		Kisumu, Kenya		Total			
Characteristic	*N* = 45		*N* = 43		*N* = 88		*N* = 277	
	N	(%)	N	(%)	N	(%)	N	(%)
**Age, years**								
Median (Interquartile range)	24	(18–30)	23	(18–29)	23	(18–30)	23	(21–26)
18–24	28	(62)	29	(67)	57	(65)	183	(66)
25–30	17	(38)	14	(33)	35	(35)	94	(34)
Currently have a primary partner	44	(98)	40	(93)	84	(96)	261	(94)
Married or cohabiting	1	(2)	20	(47)	21	(24)	79	(29)
Currently have a casual sex partner	5	(11)	12	(28)	17	(19)	50	(18)
Exchange sex ever	2	(4)	10	(23)	12	(14)	33	(12)
Parity > 0	33	(73)	31	(72)	64	(73)	216	(78)
Completed secondary school	29	(64)	18	(42)	47	(53)	143	(52)
Earns an income	8	(18)	20	(47)	28	(32)	86	(31)
**Worried contract HIV in next 12 months**								
Not at all/a little	24	(53)	31	(72)	55	(63)	179	(65)
Somewhat/very/extremely	21	(47)	12	(28)	33	(38)	98	(35)
**Method(s) ever used**^a^								
Male condom	44	(98)	40	(93)	84	(96)	255	(92)
Injectable	37	(82)	24	(56)	61	(69)	194	(70)
Implants	11	(24)	17	(40)	28	(32)	98	(35)
Pills	11	(24)	13	(30)	24	(27)	72	(26)
Intrauterine device	5	(11)	2	(5)	7	(8)	14	(5)
Female condom	3	(7)	10	(23)	13	(15)	25	(9)
Other	1	(2)	2	(5)	3	(3)	10	(4)
None	0	(0)	1	(2)	1	(1)	2	(1)
**Method(s) used at enrollment**^a,^^b^								
Male condom	29	(64)	24	(56)	53	(60)	141	(51)
Injectable	22	(49)	15	(35)	37	(42)	115	(42)
Implants	9	(20)	7	(16)	16	(18)	67	(24)
Pills	2	(4)	6	(14)	8	(9)	18	(7)
Intrauterine device	5	(11)	1	(2)	6	(7)	11	(4)
Female condom	0	(0)	4	(9)	4	(5)	8	(3)
Other	1	(2)	2	(5)	3	(3)	6	(2)
None	5	(11)	5	(12)	10	(11)	29	(11)

^a^ Can select more than one, total is more than 100%.

^b^ 35% reported dual method use currently (condoms and another contraceptive method).

### High interest in MPTs

Among those who completed the clinical study, 235 (94%) completed the questionnaire regarding preferences surrounding products for HIV and pregnancy prevention. Nearly all women (96%, *n* = 225) expressed a preference for a 2-in-1 product that prevents both pregnancy and HIV versus two separate products. There was no difference in preference by country or among those who participated in a qualitative activity (*p* = 0.75). In IDIs and FGDs, women described the appeal of increased simplicity and ease of use, reduced physical pain and decreased stress associated with remembering to use one instead of two separate products (one for each indication). One Kenyan participant summarised her view that most young women would prefer an MPT for pregnancy and HIV prevention, a method “that is simple, saves enough time … saves complications and forgetfulness and what not. So you just use one thing, that’s it” (Age 22, FGD, Kenya). Women highlighted the critical importance of a product that will protect them in unanticipated situations, including rape, condom failure and partner infidelity, and perceived MPTs as meeting this need. While most had experience with having used male condoms (a known and existing MPT), they expressed widespread dislike of condoms, primarily due to partner negotiation requirements, interference with sex and partner dislikes, implications of infidelity when asking to use male condoms and lack of trust (i.e. breakage). All three of the TRIO products were preferred over condoms, underscoring the need for new MPT delivery forms.

### Barriers to MPTs

Despite enthusiasm about the potential for new MPTs, three potential barriers to their future use consistently emerged: concerns regarding side effects, fears tied to using new biomedical technology, and personal and community-held misunderstandings and perceived stigma regarding MPT use.

### Side effects

Participants identified side effects as one of their greatest concerns regarding future MPTs. Perspectives were based on side effects constituting a primary reason for previous contraceptive method switching. Likewise, despite TRIO products being placebos, some participants linked them to negative physical reactions, some of which prompted switching among TRIO products. For example, participants linked abdominal pain to incorrect ring insertion, back pain to the injection, and nausea to the tablets. Although not a concern for most participants, several noted that a 2-in-1 product may have stronger or more “extreme” side effects relative to a single indication product.

*“The side effects may be extreme because now there’s – a drug to prevent two things. So maybe it will be strong or something*.” (Age 22, FGD, South Africa)

Interviews and FGDs also revealed misunderstandings around side effects in general that affected views toward MPTs. Some participants did not understand that side effects may not affect everyone equally. One woman was perplexed by how another participant experienced an adverse effect from the TRIO injection while she did not, and concluded the other participant must be lying:

*“There was once someone [another TRIO participant] who told me that they once injected her, I don’t know, she got swollen. But they also injected me, I never experienced that, get swollen. So I thought ‘No, I will no longer listen. People are the same, they lie’*.” (Age 19, IDI, South Africa)

Others attributed side effects to the placebo TRIO products that may have been caused by other medication they were using concurrently. One participant, for example, shared how she experienced nausea when taking two pills, one for contraception and the TRIO placebo tablet, and initially blamed the TRIO tablet for her nausea. It was only after consulting with a clinical trial doctor and taking the TRIO tablet on its own that she became convinced the nausea was actually a side effect of the contraceptive pill. Another participant linked the TRIO placebo vaginal ring with heavy menses, which may have been caused by a contraceptive product or other health condition.

*“The moment it [TRIO placebo vaginal ring] was inserted and left there, I started bleeding until the time I came back for removal. So I felt the burden because at times someone may want [sex], so how could you respond and you are ever bleeding?”* (Age 28, FGD, Kenya)

Additionally, differentiating what adverse effects were caused by the dosage forms themselves, versus side effects from drugs they may ultimately deliver, proved a particularly confusing topic. For example, some participants thought that if they experienced menses-related side effects from a contraceptive implant, they would experience the same side effects with any implant, regardless of its indication or active pharmaceutical agents.

### Lack of familiarity with new biomedical technology

Participants described their own challenges in trying the new TRIO products and thought that lack of information and fear with unfamiliar MPT would be a challenge for others in the community. They shared initial fears regarding product appearance, such as the look and size of the TRIO tablets: “*I was scared because I did not know of tablets that are colour blue. And the writings on it were also new*” (Age 26, FGD, Kenya) and “*I was scared because of the big size; it was a problem when it passes here [throat]*” (Age 19, IDI, South Africa). The vaginal ring, the most unfamiliar product, was described as raising concern prior to and during initial use. Nonetheless, for some, opinions of the ring grew more favourable simply with educational content.

*“I was worried like … the ring is a metal object that would hurt me or something of the sort. Because I was afraid before watching the [educational] video. But when I watched I was happy that I could use them as shown.*” (Age 21, FGD, Kenya)

Although many participants were open to trying new products after gaining experience with them over the one-month trial period, when reflecting at a community level, most women thought injections, a known and familiar delivery form, would be highly favoured. This was expressed directly by one South African FGD participant:

*“With the injection it’s something that we are used to. It’s common. You know why I will use the injection? It’s known, it has been around for a long time. Like even its side effects are known. And then these ones [TRIO tablets and ring], these ones will be new. You will not know how it treats this person, how it treats that person.*” (Age 26, FGD, South Africa)

### Misunderstandings

Participants shared misunderstandings that affected their use of TRIO products and, beyond the study, have implications for implementation, particularly related to product education and messaging of future MPTs to achieve high uptake and use. While several issues raised reflected misunderstandings held by TRIO participants themselves, others pointed to more widely shared beliefs to which participants did not personally adhere but they noted as prominent in their community. One misconception that emerged most consistently pertained to community members thinking TRIO participants were living with HIV due to their study participation and/or use of TRIO products. This was particularly true during periods in which daily tablets were used, with many examples of experienced stigma from partners, family and others in the community related to tablet use. For example, one participant described: “*If someone gets to see you taking these tablets they would regard them as ARVs [antiretroviral drugs] and start talking about you being on HIV treatment*” (Age 28, FGD, Kenya). One participant suggested that tablets designed to look more like contraceptive pills (e.g. pink in colour) would allow participants to describe their indication as family planning.

Another described how rumours that she was HIV positive were spread in the community:

*“The motorbike owner, who used to bring me here [clinical trial site], went back to spread rumour that nowadays I am sick [HIV positive] and taking ARVs from here and he is the one dropping me every month. This brought tension with the neighbours and my boyfriend.*” (Age 21, FGD, Kenya)

Some participants shared reservations about using contraceptives in general that also applied to MPTs. These included beliefs that contraceptives causing decreased future fertility and were not suitable for nulliparous women; religious barriers to using contraception; pressure from family or partners to have children; and restricted access to social grants (financial aid per child) from the government in South Africa.

Women also described product-specific fears that affected their adherence to and use of TRIO products. For example, two participants described a fear of the ring being pushed too deeply inside their vagina during sex, causing them to abstain from sex for the month while using the ring. One woman shared that she heard of another TRIO participant who was putting the TRIO study tablets in the toilet because she thought the blue colour of the pills meant that they were poison. Another participant misunderstood the concept of a “2-in-1” MPT product, thinking that it referred to protection for herself and her baby: “*Isn’t it that one protects the baby, the other one protects me?*” (Age 21, IDI, South Africa).

### Recommendations for roll-out/implementation of future MPTs

#### Opportunities to try MPT before deciding to use

Women strongly agreed that it was helpful to try multiple TRIO products before deciding what they preferred, and emphasised it was important to offer MPT options from which women could choose. Women responded nearly unanimously that they appreciated gaining personal experience with each product to make informed decisions about their preferred option: “*By trying the three products you were able to decide on the product that is comfortable with you*” (Age 21, IDI, Kenya). For future MPTs, some recommended offering a way to predict side effects beforehand to see if a certain product would “*rhyme with their blood [will not have side effects]*”. Others suggested a delivery model in which women were first offered an opportunity to try products for a short time first (as done in the TRIO study) before they chose one to use for a more prolonged period of time.

#### Involve men in MPT decision-making

Women expressed mixed opinions on whether male partners should be included in their choice of MPT product. Some felt that men should be included in the decision process, highlighting the need for men to receive more education about family planning:

*“I suggest that you call men as well and sensitize them. […] You see it is only us ladies being taught while they remain ignorant about most things. It is men who are in need of knowledge but there is no opportunity provided for them.*” (Age 18, FGD, Kenya)

Others thought that men would make up false stories and discourage product use, while some described existing barriers to partner involvement in contraceptive decisions and use that would also apply to MPTs. For example, one woman shared how she decided not to disclose ring use to her partner because of what she heard from other participants: “*I heard most people say that ‘no, my boyfriend is saying that this thing [ring] is irritating him.’ So I didn’t say anything when I got home*” (Age 27, FGD, South Africa). Another described her partner’s strong disapproval of her using any contraceptive method:

*“My husband does not like the idea of family planning and he does not want to hear about it. So I took a decision myself after being taught. I saw it better to join so that I should be able to choose the best product in the future for use to prevent HIV/AIDS and family planning. But I know he can never agree with this issue.*” (Age 29, FGD, Kenya)

#### Optimisation of MPT attributes

Building on their experiences with contraceptives as well as TRIO products, women recommended various attributes that could inform the optimisation of MPTs. Most women preferred a product with a long duration to minimise clinic visits and suggested that existing TRIO products should last longer (e.g. 2- to 3-month injection instead of 1 month; weekly or monthly pills instead of daily). Conversely, some women were concerned that if a product lasted too long, they would forget to continue its use over time (e.g. a monthly pill or 3 monthly injection). Some worried that a vaginal ring that lasted longer than a month would block normal bodily functions such as causing menstrual blood to build up in the body and create a smell.

Both South African and Kenyan women wanted a product that would protect in case of rape. They favoured products that are not “on demand”, would already be “in your body” (Age 29, IDI, Kenya) and would not burst during sex like a condom:

*“Sometimes thugs may attack you in the house in the estate and you can be raped […] If he rapes me, I will not be affected in any way because I will be wearing a ring, on injection or on tablets.*” (Age 26, FGD, Kenya)

Women also desired a discreet product but discussed how the level of discreetness may depend on the individual and her situation. For example, some women worried that their partner would feel the ring during sex, or that different positions during sex may make it more likely for the partner to feel the ring. They also described that method side effects could make their use obvious: heavier menstrual bleeding caused by contraceptives; lingering pain 1−2 days after an injection; palpation and visible outline with an MPT implant.

*“The Norplant [contraceptive implant] which is placed in the arm, one can easily detect it. Even the injection, someone may push the site of injection with pressure and you find yourself reacting to the pain felt.*” (Age 29, FGD, Kenya)

Most wanted a product that would enhance sexual pleasure for their male partners, or at minimum, would not interfere with sex. Indeed, though uncommon, some TRIO participants described the ring as doing so: “*He [partner] was encouraging me [to go to Setshaba for research visits]. He loves sex with a ring*” (Age 21, FGD, South Africa). One woman suggested advertising the TRIO ring as sexually enhancing, while still emphasising its efficacy:

*“Like now they [youth] are always going to the Chinese store to buy these sweets. To make it [vagina] small. […] So this thing [TRIO ring] also works like those Chinese vagina tighteners. It tightens up your vagina. And then after having sex your partner, he is going to give you lots of cash [participants laughing]. But at the same time, what I like most about the ring, is that you don’t get HIV.*” (Age 24, FGD, South Africa)

When asked how MPTs could be made more attractive to use, many suggested making the packaging more feminine, colourful, and decorative. One participant recommended that the packaging “*should be sexy. Maybe a love symbol [heart-shaped package]*” (Age 23, FGD, Kenya). Another suggested having designers create packaging like they do for menstrual pads*,* “*so that when you look at it you should feel attracted. It should make you to wanna use it*” (Age 28, FGD, South Africa). Several participants also suggested future tablets should be designed to reduce potential for confusion with antiretroviral drugs for HIV treatment and resulting stigma, and recommended they have a unique sound in the container, be smaller in size and be a different colour.

#### Novel MPT dosage forms

**Figure 2.**
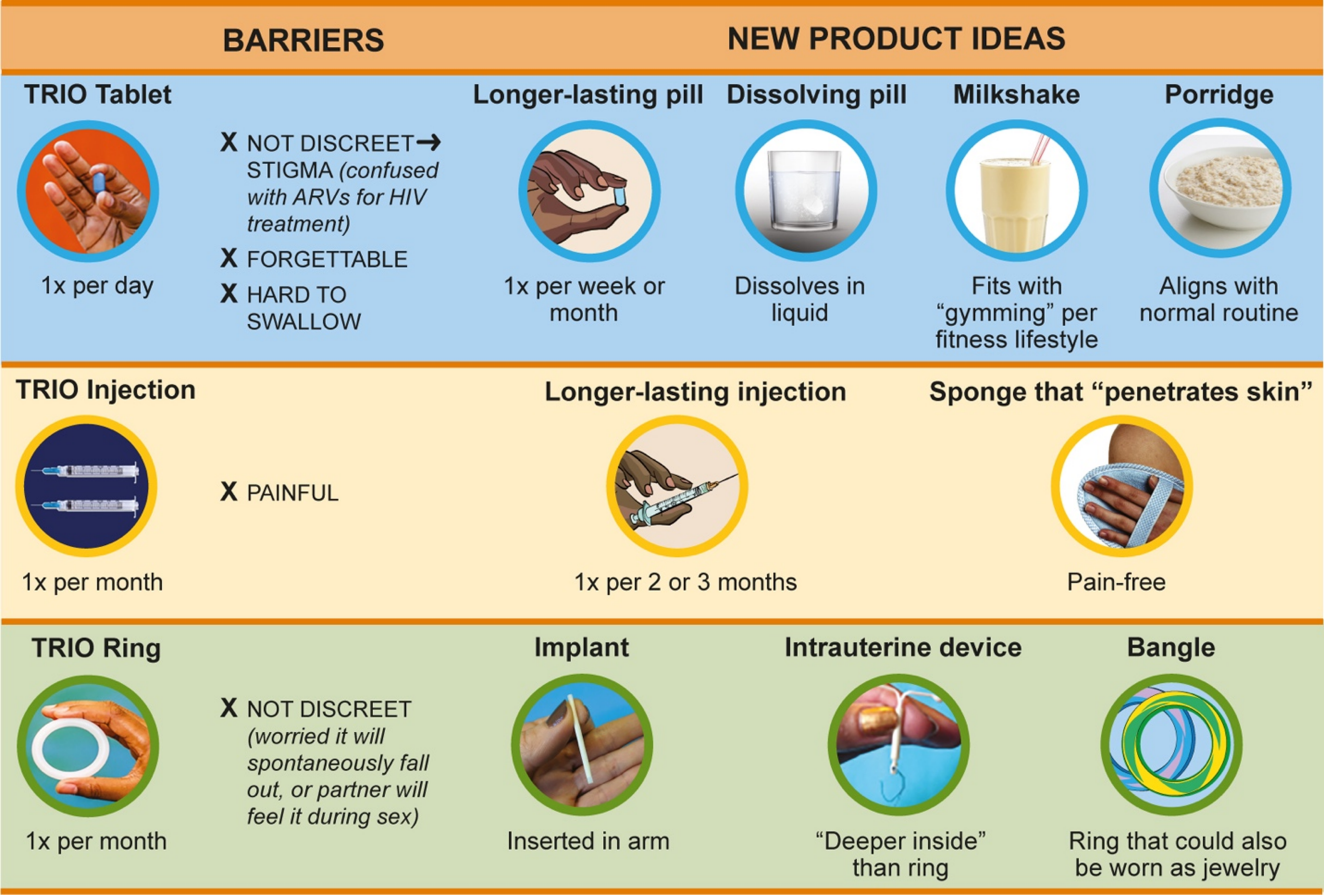
Iterations on TRIO products as suggested by women in study. Women recommended several optimised dosage forms for future MPTs to overcome challenges with TRIO products. ARV = antiretroviral

Participants recommended several improvements to overcome barriers with existing TRIO products by suggesting novel MPT dosage forms or adapting existing contraceptive delivery forms, including implants and IUDs (Figure 2). To overcome challenges with forgetting to take tablets, women recommended decreasing the dosing frequency to weekly or monthly. They also suggested making daily dosing in more discreet product forms, such as a milkshake or porridge that would integrate better into existing life patterns and, importantly, that would not be confused with an ARV for HIV treatment. To reduce injection pain, women suggested making the MPT a single injection that would last three months, or making the needle more like a “*sponge [that] should be able to penetrate into the skin*” (Age 26, FGD, South Africa). To make the ring more discreet and less likely to interfere with sex, women recommended making it like an IUD that would be inserted “deeper inside” the vagina or like an implant that would be inserted into the arm. Another suggested making a vaginal ring that looks more like jewellery and could be worn as a bangle on the arm.

## Discussion

The TRIO Study, conducted in Kenya and South Africa, highlighted young women’s keen interest in MPTs that combine pregnancy and HIV prevention. Few studies have ascertained women’s preferences and examined their perspectives on factors relevant to MPT adoption and use.^26,27^ Unique among the limited existing end-user research on MPTs, we assessed women’s views following actual use experience with three placebo products: oral tablets, vaginal ring and gluteal injections. While injections were most preferred among the three delivery forms, all of these product forms were favoured over male condoms, a widely known, existing MPT.^18^ Importantly, many barriers to MPT use that women raised were not product attribute-related or dosage form-specific, but instead addressed broader, contextual influences, such as fears tied to use of new biomedical technology, navigating use with their sexual partners, and individual and community misunderstandings regarding contraceptive and HIV prevention products. These types of barriers appear to constitute core concerns that ultimately may influence adoption and use of future products, a finding aligned with past qualitative research in South Africa on biomedical HIV prevention.^28^ Contraceptive misconceptions and challenges with obtaining partner support for their use are well documented.^29^ Likewise, health provider views toward contraceptives and HIV prevention are known to affect access and use, particularly by young women.^30^ Pre-emptively addressing potential MPT misconceptions, and developing strategies to monitor and respond to those that emerge following introduction of a new product, will be vital. Furthermore, attention to implementation strategies to introduce a new MPT in different cultural contexts is important to maximising public health benefits.^31^

Among the anticipated barriers to adoption and use of future MPTs, women primarily identified side effects. They reflected on their experiences switching contraceptive methods due to undesirable side effects and drew inferences with regard to future MPTs in envisioning possible adverse effects. Many articulated misperceptions about side effects in general, and some confused dosage-form-related side effects with drug-related side effects. This emerged, for example, in expectations that menses-related side effects experienced with a contraceptive implant would occur with any implant regardless of its active pharmaceutical agents. Given that future MPTs will contain multiple pharmaceutical agents with different side effect profiles and may present physical and/or social risks tied to the dosage form itself, there is potential for side effects, both actual and perceived, to constitute a considerable barrier to uptake and use.

Whether to involve men in women’s decisions to use MPTs was a point of debate among participants. Many articulated a need for male sensitisation and education, including to support use of family planning, a finding that is echoed as important to the adoption and use of oral PrEP and the vaginal ring for HIV prevention.^32,33^ Navigating this need in the context of intimate partner violence, which has been shown in Uganda and Kenya, for example, to affect oral PrEP adherence,^34^ requires tailored strategies. Addressing these known barriers before introducing a new product could facilitate the path for future MPT implementation. Multifaceted efforts to support long-acting contraceptive roll-out in Kenya, for example, addressed demand creation, provider support and community mobilisation to achieve increased uptake during the period 2010−2014.^35^ Interventions designed to address relationship-based barriers to the adoption of existing HIV prevention tools may likewise consider a broader reproductive health focus that includes contraceptive needs and strategies to simultaneously address both outcomes in a more integrated manner.

Young women’s recommendations for the design of future MPTs highlighted several considerations pertinent to product development. While a desire for choice among products emerged as a strong theme in TRIO,^24^ variation in duration of effectiveness within the same delivery form was proposed as an effective way to achieve this. Thus, choice was conceptualised both as a method mix composed of different product forms and variation in the duration of protection within the same form. HIV prevention and treatment regimens that consist of weekly or monthly dosing with oral tablets^36^ were attractive to young women. Interestingly, many novel product ideas proposed in FGDs align with products currently in development, such as dissolving micro-needle drug delivery systems^37^ or a transdermal patch. Likewise, multiple implants – both HIV prevention and dual protection products – are in pre-clinical and clinical development.^15,38,39^

There are several limitations to this study. First, generalisability of findings may be limited by selection bias within the study sample. Even though the qualitative sample of TRIO study participants consisted of both a randomly selected sub-sample and purposively selected participants based on their choice of product for use during the second stage of the trial, they were women who elected to enrol in a placebo clinical study examining MPT products. While we cannot assess whether their views align with general population perspectives, recruitment strategies did engage women from diverse community settings in Kisumu and Soshanguve and enrolled women who, prior to joining TRIO, had not participated in other contraceptive or HIV prevention research. It is possible that study participation, including receipt of visit reimbursements and clinical services, influenced willingness to try new technologies and tolerate negative experiences with the products. Nonetheless, pre-enrolment workshops and counselling during visits heightened knowledge about the concept of an MPT product and direct experience with the placebo products themselves gave women insight about these delivery forms, making participants well poised to comment on preferences, barriers and preferred product characteristics. Also, TRIO products were placebos which meant that participants did not gain experience with side effects of future MPTs (only those of the delivery form). However, as evidenced in the findings, participants drew on their experiences using contraceptives and clearly identified side effects as the one of main potential barriers for future use of MPTs. The importance of side effects to product preference did not come through in a discrete choice experiment choice survey conducted as part of the TRIO study.^23^

## Conclusion

MPTs that combine contraception and HIV prevention are highly desired by women and constitute an important product category in achieving a method mix that supports global goals of reduced incidence of HIV and unintended pregnancy. Indeed, global efforts to expand access to modern contraceptives in low- and middle-income countries and to increase the range of contraceptive methods offered to women demonstrate the importance of choice in meeting women’s needs and, ultimately, achieving development goals. The HIV prevention field has similarly recognised the value of providing choice to meet the diverse needs of users and their needs that change based on relationship circumstances and prevention and reproductive priorities. MPTs stand to contribute importantly to the range of options available to women and couples. Yet, early consideration of women’s preferences for products in development and perceived barriers to their use is critical to yielding products and implementation strategies that will ultimately achieve high adoption and use. The widespread interest in MPTs evidenced in this study underscores the promise of new MPT products informed by end-user needs and preferences to improve health and well-being for families and communities in many settings globally.
